# Challenges to Malaria Elimination in Ethiopia by 2030: A Review

**DOI:** 10.1155/jotm/3144857

**Published:** 2025-08-19

**Authors:** Tilahun Adugna

**Affiliations:** Debre Tabor University, P.O. Box 272, Debre Tabor, Ethiopia

**Keywords:** *Anopheles stephensi*, drug-resistance, Ethiopia, insecticide-resistance, malarial elimination, war

## Abstract

Malaria disease is a major health issue in Ethiopia, affecting three-fourths of the country's land area and more than two-thirds of the population living below 2000 m altitude. Over 42 *Anopheles* species have been identified in the country, but *Anopheles arabiensis* was the only major malaria vector, accounting for the majority of illness and mortality. However, there is a new invasive malaria vector, *Anopheles stephensi,* which is strikingly similar to the primary vector. This species transmits both *Plasmodium falciparum* and *Plasmodium vivax* and has gained resistance to all four types of insecticides, such as *A. arabiensis*. *Plasmodium falciparum* and *Plasmodium vivax* accounted for the majority of malaria cases in the country. However, these species have evolved resistance to several antimalarial medications throughout the country, adding to the burden. Furthermore, the country has just had a long civil war, which has resulted in an alarming spike in malaria cases throughout the country. Even in a few regions, such as Amhara and Oromo, the disease is spreading rapidly and beyond the control of regional health bureaus. All of these issues constitute major impediments to the country's malaria elimination aim. This article provides a comprehensive summary of the status of new and invading species, the status of insecticide-resistant malaria vectors, the ranges of drug-resistant *Plasmodium* species, the impacts of neglected *Plasmodium vivax*, and the impacts of civil war on Ethiopia's malaria elimination aim.

## 1. Introduction

Malaria is one of the most prevalent widespread tropical parasitic diseases in developing countries [1]. Ethiopia is one of the developing countries that have been seriously affected by malaria [2, 3] and still the disease is one the leading health problems in the country [4, 5]. This is mainly associated because three-fourths (75%) of the total area of country is malarious, and more than two-thirds (approximately 68%) of the total population live below 2000 m of altitude; therefore, it is considered a malarious risk area [6, 7].

Malaria is caused by the *Plasmodium* parasites, which is transmitted by the bite of a mosquito vector. In general, human being is infected by five *Plasmodium* parasite species: *Plasmodium falciparum*, *P. vivax*, *P. ovale*, *P. malariae*, and *P. knowelsi*. Of these, *P. falciparum* and *P. vivax* are the most prevalent parasites across Ethiopia, which accounted 70% and 30% of the malaria cases (morbidity and mortality), respectively [8, 9]. However, according to a number of recent research*, P. vivax* is more common in Ethiopia (69.7%, 70.41%, and 84.6%) than *P. falciparum* (30.3%, 29.59%, and 15.4%), respectively [10–12]. This is mainly because *P. vivax* malaria is a neglected disease in Ethiopia [13–15] and one of most neglected diseases in the world [13, 14, 16, 17].

Since 2005, Ethiopia has intervened against malaria by distributing long-lasting insecticidal nets (LLINs), the application of indoor residual sprayings (IRSs), introduction of artemisinin-based combination therapy (ACT) [18] with wide distribution of rapid diagnostic tests (RDTs), practices of early diagnosis, quick treatment of cases, and prevention and control of malaria among pregnant women using intermittent preventive therapy [19, 20]. As a result, recently, malaria morbidity and mortality rates have shown a significant reduction over time. The number of confirmed malaria cases and death rate decreased by 47% and 58%, respectively, between 2016 and 2019 [20]. Even though these results were achieved, in 2022, the Ethiopian federal ministry of health annual performance report (FY 2022) revealed a 66% increase in malaria cases in 2022 compared to 2019 (904,495 in 2019 vs. 1,504,405 cases in 2022) [21]. In 2023, reported cases of malaria increased by 150% and 120%, respectively, compared to the same periods in 2021 and 2022. This increment was driven by the continued effects of pandemic-related program disruptions, armed conflict, and displacement [22].

Though Ethiopia is striving to eliminate malaria by 2030, the existed challenges including the emergence of new malaria vector [23–25], the presence of insecticide resistance malaria vectors [26, 27], antimalaria drug resistance *Plasmodium* species [28, 29], long civil war [30], the presence of neglected *P. vivax* infections [14, 15], collapse of health centers, displacement and movement of the people [31], and the flourishment of extreme poverty could be the possible obstacles to materialize its goal. Thus, the primary goal of this analysis was to identify evidence that impedes Ethiopia's goal of eliminating malaria by 2030.

### 1.1. Appearance of New and an Invasive Species

In Ethiopia, over 42 species of *Anopheles* were identified [32, 33]. Of these, the major malaria vector is *Anopheles arabiensis* [34–38], whereas *A. pharoensis*, *A. funestus*, and *A. nili* are secondary vectors [18, 35, 38]. These four species were responsible for the previous malaria burden (morbidity and mortality) across the country.

However, recently there is an emergence of new and an invasive malaria vector, *A. stephensi*, in Ethiopia. This species is among one of the many essential malaria vectors in Southeast Asia, the Middle East, and the Arabian Peninsula [39]. In Africa, it was detected in various countries such as Djibouti [40], Sudan [41], Nigeria, Ghana and Somalia, Eritrea, South Sudan, United Republic of Tanzania [42], and Kenya [43]. This invasive species was first discovered in Ethiopia in 2016 using molecular and morphological approaches in Kebri Dehar, Eastern Ethiopia [23]. Then, according to WHO [39], this species has been discovered in 51 locations around the country. Afar (Semera, Gewane, and Awash Sebat Kilo), Amhara (Bati), Dire Dawa city, Somali (Jijiga, Erer Gota, Kebri Dehar, Godey, and Degehabur) [24, 25], and the southern regions (Hawassa) [44, 45] are a few of the detected areas.

*A. stephensi* transmits both *P. falciparum* and *P. vivax* [24, 25, 27, 39]. Similarly, in Ethiopia, this kind of result was found, and *A. stephensi* may be more susceptible to malaria infections [25] than *A. arabiensis*, the primary vector in Ethiopia [37], although more research is necessary to fully understand the results of the laboratory experiments carried out in Ethiopia. In Djibouti city, there was a second malaria outbreak starting in November 2013 and *P. falciparum*-infected *A. stephensi* females were among the responsible vector [40], but *A. arabiensis* was the main vector for malaria transmission in the country [46]. According to a recent report from Djibouti, *A. stephensi* has replaced *A. arabiensis* as the primary malaria vector [46]. As a result, Ethiopia would experience something similar.

Astonishingly, this species also differs from other malaria vectors because of its ability to grow and reproduce in human-made containers in clean or contaminated water [25, 47, 48] and is adapted to urban and periurban settings from rural. Moreover, *A. stephensi* is resistant to the four classes of insecticides commonly used for malaria control in Asia and India [49, 50], including Djibouti [22, 42]. Recently, various reports [26, 51] indicated the ability of *A. stephensi* resistance to the four classes of insecticides commonly used for malaria control in Ethiopia (Table 1). As a result, focus should be placed on developing innovative pesticides specifically for *A. stephensi*.

In addition to *A. stephensi*, recently, another potential malaria vector, *A. coustani*, emerged in Ethiopia, which is a known vector across Africa. In Kenya, *A. coustani* is playing a major role in outdoor transmission in various parts of the country; for example, in Taveta district [63], in Lake Victoria [64], and in Ahero and Iguhu districts [65]. This species was also reported as a secondary malaria vector in Cameroon [66]. In Madagascar, *A. arabiensis* and *A. funestus* play a major role in malaria transmission throughout the country [67]. Currently, *A. coustani* has been clearly demonstrated as playing a major role in malaria transmission, such as other principal vectors. In most areas of the country, *A. coustani* is known as a zoophilic and exophilic species [68].

In Ethiopia, various findings indicated that *A. coustani* was found to be infected by *P. vivax* in various parts of the country, for instance, in Jimma zone (Gilgel-Gibe hydroelectric dam area and Jimma town) [69–71], West Gojam zone (Bure district) [72], and different regions of the country (Arba Minch, Babile, Dilla, and Modjo) [45]. Even though these evidences are available, the Ethiopian MoH has not incriminated this species as a malaria vector till now. Until now, malaria prevention and control strategies only accounted for the four familiar vectors. Thus, Ethiopia must take lessons from Djibouti and Madagascar. All these available evidences imply that these species pose a greater challenge to controlling and then eliminating malaria in Ethiopia by 2030. Unless vector intervention strategies include this species too.

### 1.2. Insecticide-Resistant *Anopheles* Mosquitoes

In Ethiopia, the major malaria vector is *A. arabiensis* [36, 37], whereas *A. pharoensis*, *A. funestus*, and *A. nili* are secondary vectors [18, 38]. These malaria vectors have been prevented by using LLINs and IRSs [19, 20]. However, the emergence and spread of insecticide resistance in the major malaria vector of Ethiopia may compromise the current IRS- or LLIN-based malaria control interventions and thus threaten malaria control and elimination efforts [54, 56, 73].

In different regions of the nation, *A. arabiensis* has developed resistance to the four kinds of insecticides (pyrethroids, organophosphates, carbamates, and organochlorines) (Table 1) [56–58, 61]. Furthermore, *A. stephensi*, the new invasive species, is relatively new to Ethiopia; nonetheless, it has been noted that this species is resistant to the majority of pesticides used for IRSs and LLINs from various Ethiopian regions [26, 27, 51] (Table 1). Using all of these realities, it is reasonable to conclude that the current adult malaria vector control approaches are significantly less successful, and that new control methods, such as larval source management, should be used [27, 74]. Parallel production of innovative pesticides is required to manage pesticide-resistant malaria vectors.

### 1.3. Drug-Resistant *Plasmodium* Species

Despite the fact that malaria is being eradicated in various countries throughout the world, it remains a fatal disease in sub-Saharan Africa, killing over half a million people each year, the majority of whom are young children. This is primarily due to the presence of a very conducive climate for the spread of malaria year-round, insufficient healthcare systems that make it difficult for infected people to receive the treatment they require, and the growing drug resistance of *Plasmodium* species [75].

Resistance is the “ability of a parasite strain to survive and/or multiply despite the proper administration and absorption of an antimalarial medicine in the dose normally recommended but within the tolerance of the subject” [76]. The emergence of antimalarial drug-resistant *Plasmodium* parasites hampered the management and control of the disease [77, 78]. *P. falciparum*, the parasite that causes the most severe form of malaria, has become resistant to every antimalarial medication now on the market, including the derivatives of artemisinin. In 1998, resistance to chloroquine (CQ) was documented [79, 80]. However, CQ remains the first-line treatment for *P. vivax* malaria in many parts of the world [16], including the majority of Ethiopia [81–83]. This is because it is chemically stable, inexpensive, effective against all forms of malaria, and low in toxicity. Furthermore, the medicine's maker can readily store and supply QC even under adverse weather conditions [84]. All of these advantages made QC more desirable.

In sub-Saharan Africa, more than 90% deaths were caused by *P. falciparum*, which was treated by QC [75]; however, the emergence and spread of CQ-resistant *Plasmodium falciparum* (CRPF) occurred in Africa only about 2 decades after the first observations from South America (Colombia, Venezuela, Brazil in 1960) and Southeast Asia (Thailand/Kampuchea in 1961) [85]. This was the moment at which Africa was facing one of the greatest public health challenges [86, 87]. Similarly, CQ-resistant *P. vivax* (CRPV) was first reported in 1989 from Papua New Guinea and then Indonesia and Oceania [88].

In Ethiopia, CRPF and CRPV were reported for the first time in Debre Zeit in 1993/1994 [89]. Following this report, there are huge numbers of records about the presence of CRPF and CRPV though CRPF is most resistant to wide varieties of drugs across the country [28, 29] (Table 2). If the emergence of drug-resistance is not managed well soon, there will be increased malaria morbidity and mortality that could reach epidemic proportions in an endemic area [93, 94]. Moreover, there will be increased costs for malaria control programs because replacement antimalarial due to resistance are usually more expensive than the original drugs [95]. Therefore, preventing the future development of resistance and maintaining the effectiveness of vector control interventions are crucial to maintaining considerable advances in malaria control and the success of the malaria eradication program [96]. The introduction of novel, potent antimalarial drugs or their administration can accomplish this [97].

### 1.4. Neglected *Plasmodium vivax*

*P. vivax* malaria is one of the most neglected diseases in the world [13, 14, 16, 17, 98]. However, in recent years, the significance of *P. vivax* as a threat to global health has increased, which is connected with its re-emergence, increased transmission, overall burden, economic impact, and severity of disease [14, 16]. Globally, between 2.5 and 2.6 billion people are estimated to be at risk of the disease [99], and the number of annual infections ranges between 130 and 435 million [13, 14]. Reports of significant morbidity and mortality have demonstrated that this form of malaria is less benign than previously believed [13, 14]. The parasite has returned to previously disease-free areas too [16].

In particular to Ethiopia, there are four types of *Plasmodium* species: *P. falciparum*, *P. vivax*, *P. malariae*, and *P. ovale* [38, 100], but *P. falciparum* and *P. vivax* are the most important parasites [6, 101] and found in almost all parts of the country, extending 3000 m above sea level. Of these species, *P. falciparum* is the principal malaria case in sub-Saharan Africa [39], which is associated with severe morbidity and mortality and its capacity to evolve mechanisms to resist and tolerate very vital antimalaria drugs.

Because of this, the Ethiopian MoH has given only attention to *P. falciparum* because it is the most virulent species that has caused severe malaria and death in previous years [6, 34, 102–104] and widespread (endemic) nature across the country [18]. Based on all laboratory-confirmed cases, Ethiopia had a larger proportion of *P. falciparum* (70%) than *P. vivax* (30%) [105]. In some districts, *P. falciparum* was more abundant than *P. vivax* over time [106–109].

Taking these facts into account, the Ethiopian MoH introduced and scaled up ACTs as the first-line drug for treating *P. falciparum* malaria in 2004, as well as RDTs to improve diagnosis, and the implementation of LLINs and IRSs as a method of preventing parasite transmission from mosquitos to people [18]. Because of these, malaria morbidity and mortality rates have decreased significantly over time. Between 2016 and 2019, the death rate and confirmed malaria cases reduced by 58% and 47%, respectively [20].

Recent research, however, revealed that *P. vivax* outnumbered *P. falciparum* in a number of the nation's districts [110–114]. Further research studies confirmed the existing fact that *P. vivax* was more common than *P. falciparum* in Butajira (*P. vivax*, 62.5%) [111], Hallaba (*P. vivax*, 70.41%) [11], Adim Tullu (*P. vivax*, 84.6%) [12], and Wolkite districts (*P. vivax*, 69.7%) [10]. The underestimation or absence of *P. vivax* infections as a neglected public health issue in Ethiopia has been linked to these results [13–15]. The low-density blood stage parasitemia [115, 116] and the Duffy negativity phenotype (a high degree of protection against *P. vivax*) are linked to the underestimate of this parasite [117, 118]. Lower parasitemia is typically associated with a higher risk of misdiagnosis, particularly by unskilled microscopists who lack the comprehensive training necessary to recognize *P. vivax* [119]. Thus, in the future, *P. vivax*, like *P. falciparum*, will have a significant impact on health and the economy in the country, extending the country's malaria elimination target.

Totally, *P. vivax* is a major health concern in Ethiopia compared to other east African nations [120]. Therefore, control of *P. vivax* transmission is possible by increased research efforts to understand the biology of *P. vivax* exoerythrocytic stages, the mechanisms of resistance of *P. vivax* to antimalarial and develop new-generation antimalarial with activity against hypnozoites, the behavior of local anopheline vectors and malaria transmission patterns to optimize vector control using insecticides and insecticide treated materials, and develop anti-infection and transmission-blocking vaccines for *P. vivax* [16].

### 1.5. Civil War

When a civil war or a major societal conflict breaks out, people often flee to avoid the consequences. There are numerous reasons for the rise in malaria cases as a result of forced migration. First and foremost, the majority of people fleeing urban areas are not immune to malaria. Second, malaria is more prevalent in rural regions because the vector can live longer in a suitable environment. Furthermore, the anarchic condition induced by this social disturbance and the military importance on paved roads drive people through unfamiliar rural areas, dumps, and forests in order to escape areas of military activity, so actually aiding to facilitate its occurrence [121] because people are without any protection against the repeated *Anopheles* mosquito bites. Furthermore, war/conflict has been shown to significantly increase the risk of malaria-related mortality by raising infection risk and reducing healthcare delivery [122–124]. Different review reaffirmed this reality too [123, 125].

During world war first (WWI), impressive resurgence in malaria numbers were recorded for those malaria areas, e.g., India and sub-Saharan Africa [126]. It was exacerbated by the immigration of nonimmune European soldiers further created ideal conditions for the explosive epidemics which occurred [125]. In Tigray region, Ethiopia, there was a war between the Ethiopian federal forces and Tigray liberation forces in the early November 2020 [127], resulting in an increased malaria cases across the region [30]. Most health institutions in the region had stopped functioning because all health professionals were engaged in the war and others assigned only to treat war victims [30]. After the end of the war, the farmers were unable to harvest the expected crop, and currently, most of the rural communities are starving and expect donations from the international and external communities. All these challenges have increased the burden of malaria in the region.

In the Amhara region, Ethiopia, a fight between federal forces and ‘Fano' began in August 2023. Though the war's consequences were poorly measured, and it resulted in a number of unforeseen outcomes: (1) The government security forces deliberately wrecked over 1700 health establishments across the region. For example, approximately 200 healthcare facilities (including pharmaceuticals) in the South Gondar zone were stolen and damaged by government security forces (Ethiopian military, police, and militia) and are now purposely malfunctioning [128]. This deliberate destruction has left countless individuals without access to essential medical services, exacerbating the humanitarian crisis in the region. As a result, many communities are now facing increased health risks and a lack of adequate care, further deepening the impact of the ongoing conflict. (2) Referral hospitals are typically limited to treating war victims of Ethiopian government troops only. Recently (since July 2024), the Amhara health institution announced that hospitals have ceased operations due to a lack of operating funds [129]. (3) Since January 2024, the Amhara region state is avoiding the opening of new clinics because the government believes that these health facilities may treat ‘Fano' forces [130]. As a result, many individuals are left without the necessary interventions to prevent and treat malaria, exacerbating the health crisis in the Amhara region.

Moreover, Human Rights Watch reports that in the Amhara region, government security forces (Ethiopian military, police, and militia) have killed health workers, tortured, threatened, and assaulted doctors and nurses, wrongfully arrested patients, looted and destroyed medical supplies, and abused healthcare facilities. They have attacked ambulances, including at least one suspected drone strike [31]. Because of this, a recent report from the Amhara Region Health Bureau revealed that, as a result of the ongoing civil war, over a million cases of malaria were reported throughout the region between January and June 2024 [129]. The Amhara region's health department has declared that unchecked malaria is spreading widely throughout it. Moreover, Addis Standard also reported the presence of an alarmingly increasing new malaria infection and death rate in Oromia's West Wollega Zone as of July 2024 [131]. Similarly, over 2.1 million cases of malaria, including 371 deaths in the Oromia (35%), Amhara (22%), Southwest (13%), and South (10%) regions of Ethiopia, were reported between January and June 2024 [132]. This was primarily due to the country's continuous civil war. However, both reports underestimate the actual malaria infection rates/cases because the “Fano” and Oromo liberation forces (OLFs) control more than 90% of the Amhara area and a portion of the Oromia region, respectively. Malaria experts are not present in these areas, and government and regional malaria officials have only collected data from zonal cities and towns in a few districts.

Furthermore, in the other regions of Ethiopia, there has been an ongoing war between the Afar and Somila regions, between the Afar and Oromia regions, and sometimes between the Somila and Oromia regions [133]. As a result, approximately 5.5 million people have been displaced in Ethiopia owing to conflict, violence, drought, and flooding [127, 133], and these factors have facilitated people's relocation. Potentially, people movement is the most important factor in the transmission of malaria [121]. All of this information suggests that meeting malaria elimination targets by 2030 is unattainable. This underscores the urgent need for comprehensive strategies that address not only malaria prevention and treatment but also the root causes of displacement and conflict. Without a concerted effort to stabilize these regions and improve living conditions, achieving malaria elimination will remain a distant goal.

### 1.6. Miscellaneous Factors

Other obstacles to malaria elimination in Ethiopia include a lack of well-trained people (entomological and parasitological), surveillance, inadequate demarcation of malaria zones, and insufficient reporting of real cases. For example, the invasion and spread of *A. stephensi* in eastern Africa point to greater inadequacies in vector surveillance and management. Many countries, including those affected by the invasion, still lack the capacity and resources to conduct effective entomological assessments, such as monitoring for invasive species, understanding insecticide resistance profiles, and investigating pathogen transmission by prevailing vector populations [134, 135]. This lack of capacity not only hinders immediate response efforts but also compromises long-term strategies for malaria control and elimination.

Malaria reporting is less accurate in Ethiopia's border region with Somalia due to insufficient capacity and better surveillance methods, which affects malaria elimination plans [136]. Although the Ethiopian MoH has designated locations over 2000 m as malaria-free zones [136], they are not completely malaria-free at the district level [81, 109, 137–139]. Unless reducing all these limitations, the burden of malaria would continue to be the top health issue in the country and jeopardize the malaria elimination goal in general.

## 2. Conclusions

Ethiopia has set a 2030 deadline to eradicate malaria by taking into account its past recorded results and efforts. Preventing further development of resistance and maintaining the efficacy of vector control strategies are crucial for maintaining notable progress in malaria control and the success of malaria eradication initiatives [96]. The nation's present effort to eradicate malaria, however, is having the most difficulties. Among the most difficult are the emergence of novel and invasive *A. stephensi*, the existence of *Plasmodium* species resistant to antimalarial drugs (*P. falciparum* and *P. vivax*), and the presence of insecticide-resistant malaria vectors (*A. arabiensis* and *A. stephensi*) used for IRSs and LLINs. Furthermore, efforts to prevent and treat malaria as well as to eradicate it altogether have been hampered by the ongoing civil war between federal forces and the Tigray region and between the Amhara region and the federal. Additionally, *P. vivax* is a parasite that is often overlooked in Ethiopia, where reports of its prevalence are higher than those of *P. falciparum*. In addition to these factors, the government lacks qualified entomological and parasitological specialists; poor monitoring strategies, a poor malaria zone demarcation, and a lack of reporting of actual cases hinder the nation's efforts to eradicate malaria.

As a result, control tactics must cope with a complex vector system that includes a few major vectors and several secondary vectors whose effects on transmission vary by area [16]. The present malaria prevention and control approaches consider *A. stephensi* and *A. coustani*. A multicenter national assessment of these species is needed to better understand their range and role in malaria transmission. Though CQ is the recommended treatment for *P. vivax* malaria in Ethiopia, a multicenter national assessment is needed to determine the extent of *P. vivax* resistance to CQ. Furthermore, to manage antimalarial drug resistance in Africa, WHO established four essential pillars, which should be implemented [140]. Overall, battling antimalarial medication and pesticide resistance requires new effective medications, and donors must be encouraged to create an active antimalarial drug [97]. Overall, combating antimalarial medication and pesticide resistance necessitates new effective medicines, and donors have to be fostered the development of an active antimalarial drug [97]. The government also resolves the existing conflict by taking necessary political measures. If so, Ethiopia will be able to achieve its malaria elimination goal by 2030.

## 3. Limitation of This Review

Civil war, forced displacement, drought, flooding, and landslides occur throughout the country. These reasons resulted in almost 5.5 million displaced persons around the country. However, their impact on the country's malaria prevalence rate was not precisely measured. Specifically, the effects of the continuing civil war across the country have not been well analyzed. This could be a potential limitation of this review.

## Figures and Tables

**Table 1:** Types of pesticide-resistant *Anopheles* species in Ethiopia.

Names of insecticide chemicals used in Ethiopia	Insecticide-resistant *Anopheles* species in Ethiopia	Districts of Ethiopia, where resistance reported	Reported years	References (cited in)
DDT, permethrin	*A. arabiensis*s	Melka Worer Metehara	2003	[52]
DDT, malathion, permethrin, deltamethrin	*A. arabiensis*	Wondogenet/Ado/, Alaba, Gantasira/Araba Minch/, Dangora, /Aletachuko/, Andasa and Yenesa/Bahirdar/	2011	[53]
Bendiocarb, propoxur, malathion, DDT	*A. arabiensis*	Bahir Dar Zuria, Jabi Tehnan, Tach Armacho, Mirab Abaya, Boloso Sore and Soddo Zuria	2013	[54]
DDT, deltamethrin	*A. arabiensis*	Chewaka, Ziway-Dugda, Asendabo, Halaba, Alamata, Amibara, Lare	2017	[55]
Deltamethrin	*A. arabiensis*	Ghibe river valley, Sodere hot springs, Gorgora, Jimma, TiroAfeta, OmoNada, Kerssa, Itang	2010, 2012, 2022	[56–58]
Alpha-cypermethrin	*A. arabiensis*	Itang, shores of Lake Abaya, along the Harrae river	2013, 2022	[58, 59]
Bendiocarb	*A. arabiensis*	Jimma, TiroAfeta, OmoNada, Kerssa	2012, 2015	[57, 60]
Propoxur	*A. arabiensis*	Jimma, TiroAfeta, OmoNada, Kerssa	2010, 2013	[56, 57]
DDT, deltamethrin.	*A. arabiensis*	Bahir Dar Zuria, Jabi Tehnan,Tach Armacho, Mirab Abaya, Boloso Sore, Soddo Zuria	2013	[54]
DDT, pyrethroids	*A. arabiensis*	Mankush, Chewaka, Tolay, Asendabo, Bako, Sodore, Shellemele, Goro, Guba Hora	2017	[61]
Pyrethroids	*A*. *arabiensis*, *A*. *amharicus*	Arjo Didessa	2022	[62]
Bendiocarb, propoxur, DDT, malathion, permethrin	*A. stephensi*	Kebri Dehar	2020	[26]
Pyrethroids, carbamates, organophosphates	*A. stephensi*	Dire Dawa, Kebridehar, Awash Sebat Kilo, Metehara towns	2021	[27]
Bendiocarb, pirimiphos-methyl, permethrin, propoxur, alpha-cypermethrin, deltamethrin	*A. stephensi*	Awash Subah Kilo town, Haro Adi village around Metehara	2023	[51]

**Table 2:** Antimalaria drug-resistant *Plasmodium* species in Ethiopia.

Names of antimalaria drugs	Types of drug-resistant *Plasmodium* species	Reported years	References (cited in)
Chloroquine (CQ)	*P. falciparum* and *P. vivax*	1996	[89]

CQ and sulfadoxine-pyrimethamine (SP)	*P. falciparum*	2005	[28]

CQ and SP	*P. falciparum*	2006	[29]
SP	*P. vivax*	2006	

CQ	*P. vivax*		[90]

CQ and artemether-lumefantrine/AL/	*P. vivax*	2011	[91]

Artemisinin-based combination therapy (ACT)	*P. falciparum*	2024	[92]

## Data Availability

Data sharing does not apply to this article because no new data were generated.

## Nomenclature

IRSs: Indoor residual sprayings
LLINs: Long-lasting insecticidal nets
ACT: Artemisinin-based combination therapy
RDTs: Rapid diagnostic tests
PMI: President of malaria imitative
DDT: Organochlorine dichlorodiphenyl trichloroethane
CQ: Chloroquine
CRPF: Chloroquine-resistant *Plasmodium falciparum*
CRPV: Chloroquine-resistant *Plasmodium vivax*
SP: Sulfadoxine-pyrimethamine
AL: Artemether-lumefantrine
MoH: Ministry of health
OLFs: Oromo liberation forces
WHO: World Health Organization
WWI: World war first
IOM: International organization for migration
OCHA: United Nations office for the coordination of humanitarian affairs
RBM: Roll black malaria
